# Mapping changes in women's visual functions during the menstrual cycle: narrative review

**DOI:** 10.1590/1516-3180.2020.0474.R2.03052021

**Published:** 2021-11-15

**Authors:** Bruna Gabrielli Damascena de Figueiredo, Maria Thalita Cardoso Rezende, Natanael Antonio dos Santos, Michael Jackson Oliveira de Andrade

**Affiliations:** I Psychology Student, Neurosciences and Behavior Laboratory, Department of Psychology Perception, Universidade Federal da Paraiba (UFPB), João Pessoa (PB), Brazil.; II Psychology Student, Neurosciences and Behavior Laboratory, Department of Psychology Perception, Universidade Federal da Paraiba (UFPB), João Pessoa (PB), Brazil.; III PhD. Full Professor, Neurosciences and Behavior Laboratory, Department of Psychology Perception, Universidade Federal da Paraiba (UFPB), João Pessoa (PB), Brazil.; IV PhD. Associate Professor, Laboratory of Neuroscience, Chronobiology and Sleep Psychology, Department of Psychology, Universidade do Estado de Minas Gerais (UEMG), Divinópolis (MG), Brazil.

**Keywords:** Vision disorders, Menstrual cycle, Progesterone, Narrative review [publication type], Healthy women, Estrogen, Basic visual functions, Visual contrast

## Abstract

**BACKGROUND:**

This article systematically updates the literature on changes in visual functions during the phases of the normal menstrual cycle in women.

**OBJECTIVES:**

To update Guttridge's 1994 review of visual structures and functions associated with the menstrual cycle and broaden the search through psychophysical, neuroimaging and neurobehavioral measurements covering 1994-2020.

**DESIGN AND SETTING:**

Narrative review conducted in a neurosciences and behavior laboratory in Brazil.

**METHODS:**

The PubMed, Cochrane Clinical Answers and Google Scholar databases were searched. After screening and applying the eligibility criteria, 32 articles were examined. Through this analysis, the following information was extracted: (1) geographical distribution of the study; (2) sample size (according to age and phase of the menstrual cycle); (3) type of measurements according to psychophysical, neuroimaging and neurobehavioral instruments; (4) vision testing model; (5) visual subcategory evaluated; (6) categories of processed visual stimuli; and (7) main findings.

**RESULTS:**

The menstrual phases give rise to significant changes in visual functions, including in relation to orientation and spatial attention, visual campimetry and visual sensitivity. These relate specifically to the follicular and luteal phases.

**CONCLUSIONS:**

These findings theoretically expand the effects of menstrual cycles on visual functions found by Guttridge (1994). Despite some inconsistencies in the studies analyzed, it was found that visual processing during the follicular and luteal phases of the normal menstrual cycle of healthy women can explain physiological, cognitive, behavioral and social modulations.

## INTRODUCTION

The menstrual cycle of women is tightly controlled by endocrine, autocrine and paracrine factors that regulate ovarian follicular development, ovulation, luteinization, luteolysis and endometrial remodeling.^1^ The female human reproductive cycle is characterized by a cycle duration ranging from 26 to 35 days and can be described in terms of ovarian and uterine or menstrual cycles that are controlled by the hypothalamic-pituitary-ovary system.^2^

The ovarian cycle and its hormonal regulation consist of three phases: follicular (days 1 to 10), ovulatory (days 11-14) and luteal (days 15-28). Didactically, the menstrual cycle is considered to be the interval between the first day of menstruation and the beginning of the next. Thus, 24 hours before the end of the menstrual cycle, the levels of estrogen and progesterone decrease, which results in menstrual bleeding and starts a new cycle.^3^ The follicular phase, also known as the early or menstrual follicular phase, is characterized by low levels of estrogen and progesterone. The ovulatory phase is characterized by high levels of estrogen and low levels of progesterone. In the luteal phase, also known as late luteal or premenstrual phase, high levels of estrogen and progesterone occur.

It is important to note that estrogen levels can alter the functioning of the central nervous system. Hormonal changes in the premenstrual phase can compromise the control of homeostasis and labyrinthine fluids, thus generating changes to balance and hearing. Moreover, the sensory thresholds of taste, hearing, pain, smell and vision vary in their functional performance.^4^

The variability of inhibitory neurotransmitters that bind to visual receptors during cyclical hormonal variations can alter visual sensitivity.^5^ Guttridge reviewed the literature on the ophthalmological and functional changes that occur in the eye system during the normal human menstrual cycle.^6^ The main findings from that review pointed to variations in thickness, curvature, corneal sensitivity, intraocular pressure, maturation of the connective membrane and production and secretion of vicarious hemorrhagic tears in the eye. Among the functional measurements, changes to flashlight thresholds, detection and discrimination of visual stimuli, visual acuity and color vision can be highlighted.^7^ Most of the studies discussed had an ophthalmological or clinical focus and did not explore the psychophysical, neurobehavioral and electrophysiological functioning of vision.^6,8^

Although several conclusions about ophthalmic function performance during the phases of the normal menstrual cycle were reached in previous studies, these data presented some conflicting features, such as with regard to increased visual sensitivity in the premenstrual period.^6^ Thus, there is a need to update the data sources on visual function performance during the menstrual cycle.

## OBJECTIVES

The phases of the menstrual cycle may be clinically significant in relation to the mechanisms of visual processing, as this may facilitate understanding of the mechanisms underlying visual functions. To this end, the aim of this study was to investigate the methodological practices involved in studies on the normal menstrual cycle, regarding processing of the visual system of healthy women and the underlying visual mechanisms.

## METHODS

We carried out an up-to-date search for studies that describe the use of psychophysical, neuroimaging and neurobehavioral tools for analyzing the visual functions of women during the normal menstrual cycle. Our search covered the period from the Guttridge study conducted in 1994, up to 2020. The following databases were used: PubMed, Cochrane Clinical Answers and Google Scholar. The following terms were used: “*visual functions*” OR “*visual system*” OR “*visual perception*” OR “*vision*” AND “*menstrual cycle*” OR “*estrous cycle*” OR “*menstrual period*” OR “*ovarian cycle*”. The reference lists of the studies found were also reviewed to identify any additional studies. This systematic review was reported in accordance with the Preferred Reporting Items for Systematic Reviews and Meta-Analyses (PRISMA) guidelines. The keywords were chosen even in the absence of the specific Medical Subject Heading (MeSH) term, in order to prioritize sensitivity over specificity.

### Research strategy

During the first screening, two reviewers (BG and MT) evaluated the titles and abstracts of each study and excluded those that did not meet the eligibility criteria highlighted below. For each study selected, the full article was read and evaluated to verify whether it met the criteria that had been established. If any disagreements arose, a third evaluator (MA) was contacted. At the screening stage, all titles were analyzed, and studies that were considered important for the topic addressed were selected. Then, the abstracts corresponding to the titles that had been selected were read. Studies that were considered irrelevant to the objective of the present study were excluded. Lastly, the articles thus selected were read in full and further exclusions were made in accordance with the eligibility criteria.

### Eligibility criteria

The inclusion criteria were that the studies need to have the following characteristics: (1) randomized clinical trials, quasi-randomized clinical trials, case-control or cohort studies; (2) studies that referred to the normal menstrual cycle; (3) adult and/or healthy women were the participants; (4) visual functions were evaluated; (5) psychophysical, neuroimaging and neurobehavioral instruments were used; (6) full article written in English; and (7) published between 1994 and 2020. The exclusion criteria were the following situations: (1) studies that referred to abnormal menstrual cycles; (2) only visual structures were evaluated; (3) review studies; (4) studies that were carried out during pregnancy or menopause; (5) studies exclusively on use of contraceptives; and (6) letters, editorials, systematic reviews and bibliographic reviews. Studies that showed insufficient information, such as in the statistical analysis, or that showed incomplete procedures, were also excluded.

### Quality assessment

The articles were evaluated based on internal validity (e.g. selection bias, performance bias, friction measurement bias and reports) and the validity construct (e.g. suitability of the operational criteria used). In general, the quality of the evidence from the studies was assessed using three main measurements: (1) limitations (poor design, for example); (2) consistency of results; and (3) accuracy (ability to generalize findings and provide sufficient data). Studies that failed with regard to these points were not added or selected.

### Data extraction

After assessing the articles in accordance with the pre-established criteria, the following information was extracted for analysis purposes: (1) geographical distribution of the study; (2) sample size (according to age and phase of the menstrual cycle); (3) type of measurements according to the psychophysical, neuroimaging and neurobehavioral instruments; (4) vision testing model; (5) visual subcategory evaluated; (6) categories of processed visual stimuli; and (7) main findings. “NS” (not specified) was used in the tables to denote situations of insufficient information from the studies to determine the data, according to the category.

## RESULTS

**Figure 1:**
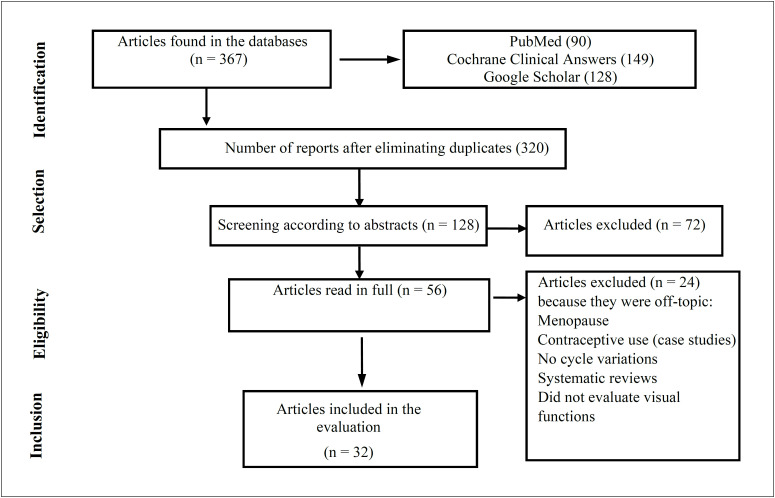
Results from database searches and selection process.

Figure 1 shows a detailed flow chart of the study selection process. The tabulation showed that a total of 320 potentially eligible articles were found. Many articles were found to be incompatible with the purpose of the present study and/or featured women with irregular menstrual cycles, women using contraceptives, pregnant and lactating women, or women entering menopause. In addition, some studies did not directly assess measurements of visual function during the different phases of the normal menstrual cycle. Thus, after screening and applying the eligibility criteria, a total of 32 articles were examined.

Table 1 summarizes the articles according to the psychophysical methodological criteria. A total of 12 articles were found to fit within the criteria that had been established.

**Table 1:** Description of the results according to the psychophysical method

Authors (year)	Country	Sample (phases of the cycle)	Method	Testing	Visual task	Stimuli	Results
Darlington, Ross, King & Smith (2001)^24^	New Zealand	12 women aged 21.9 ± 4.0 years (menstrual, follicular, half-cycle and luteal phases)	P	NS	Eye movement	Oculogram and optokinetic (OKR)	There was no difference in eye movement speed
Apaydin, Akar, Akar, Zorlu & Ozer (2004)^34^	Turkey	93 eyes of women in the follicular and luteal phases: 45 eyes of women with diabetes mellitus (34.9 ± 4.7 years) and 48 healthy controls (36.2 ± 5.2 years)	P	M (left eye recording)	Visual field	Humphrey Field Analyzer II (upper temporal, lower temporal, upper nasal and lower nasal)	There were no differences between women with and without diabetes. Women with diabetes had lower sensitivity in the visual field in the luteal phase
Eisner, Burke & Toomey (2004)^28^	United States	4 women aged 21–29 years (menstrual, follicular and luteal phases)	P	M	Visual sensitivity	Temporal stimulus of 1.5 Hz square wave (wavelengths: 440, 460, 490, 510, 540, 580 and 640 nm)	No variation in visual sensitivity
Hausmann (2005)^23^	New Zealand	24 women aged 33.93 ± 10.1 years (menstrual, follicular and luteal phases); 14 men aged 26.96 ± 6.19 years	P	NS	Spatial orientation	Line bisection task (17 horizontal lines)	Greater activation of spatial hemispheric asymmetry in the luteal phase
Giuffrè, Rosa & Fiorino (2007)^31^	Italy	15 women aged 25 ± 4.0 years (3 evaluation phases: menstrual, ovulatory and luteal)	P	M (left-eye and right-eye recording)	Visual acuity, luminance contrast and color view	Vertical sine grids; Farnsworth-Munsell 100-hue test	There were no differences in visual acuity or luminance contrast; greater color vision in the ovulatory phase
Akara et al. (2013)^18^	Turkey	59 eyes of women (follicular and luteal phases) 54 eyes of men	P	Monocular	Visual field	Humphrey Field Analyzer II (upper temporal, lower temporal, upper nasal and lower nasal); Ishiara's color test	Smaller auto-tinted perimeter in visual field sensitivity in the luteal phase
Kumar, Mufti & Kisan (2013)^21^	India	30 women aged 21.33 ± 2.3 years (menstrual, follicular and luteal phases)	P	NS	Visual (TRV) and auditory (TRA) reaction time	Reaction time after the presentation of stimuli (green light; 4,000 Hz)	TRV and TRA were higher during the luteal phase and lower during the follicular phase
Danilenko & Sergeeva (2015)^43^	Russia	16 women aged 27.5 ± 1.9 years (follicular and mid-cycle phases)	P	NS	Visual sensitivity	Lumie SADlight with LEDs; light flashes of wavelengths: blue (480 nm) and red (651 nm)	Greater visual sensitivity at 480 nm during the follicular phase
Abdul et al. (2015)^19^	United Kingdom	30 women in the follicular phase (19.72 ± 1.20 years); 24 women in the luteal phase; 30 men (20.16 ± 1.02 years)	P	Binocular	Spatial orientation	CRFT system (previous mechanical rod and frame test); line stimuli	Largest error in the spatial orientation of verticality for women in the luteal phase
Sudheer, Jagadeesan & Kammar (2016)^33^	India	50 women aged 20-25 years (3 phases of evaluation: menstrual, ovulatory, and luteal)	P	NS	Visual (TRV) and auditory (TRA) reaction time	Reaction time after presentation of stimuli (flash/sound)	TRV and TRA were higher during the menstrual phase and lower during the luteal phase
Sy et al. (2016)^11^	United States	16 women; mean age 22 years (follicular and luteal phases)	P	M (left/right eye recording)	Binocularity	Psychophysics toolbox; circular sinusoidal grid stimuli (4.5 and 30%; 1.48 in diameter)	There was no significant difference in binocularity or temporal dynamics
Webb, Hibbard & O’Gorman (2018)^20^	United Kingdom	21 women aged 21.6 ± 3.8 years (menstrual, ovulatory and luteal phases); 14 women who used contraceptives; 14 men	P	NS	Luminance contrast	Stimuli were Gabor's sinusoidal grids with spatial frequencies of 1, 4 and 16 cpd	There was no difference in luminance contrast according to hormonal shifts

P = psychophysical; NS = not specified; M = monocular; cpd = cycles per degree.

Table 2 summarizes the articles according to the neuroimaging methodological criteria (studies with spatial and temporal paradigms). A total of six articles were found to fit within the criteria that had been established.

**Table 2:** Description of the results according to the neuroimaging method

Authors (year)	Country	Sample (phases of the cycle)	Method	Testing	Visual task	Test and stimuli	Results
Yılmaz, Erkin, Mavioglu & Sungurtekin (1998)^14^	Turkey	30 women aged 29.6 ± 6.4 years (4 evaluation phases: menstrual, follicular, ovulatory and luteal)	N (VEP)	M (left/right eye recording)	Visual electrophysiology	Visual evoked potential (reverse pattern); P100 latency and visual range (Oz)	Higher mean P100 amplitude during the ovulatory phase
Krug et al. (2007)^13^	Germany	11 women (in the menstrual, ovulatory and luteal phases); mean age 25.3 years	N (VEP)	NS	Visual electrophysiology	Social stimuli (sexy man, baby, people, woman); N1, P2, P3, LPC, SW (700-900 ms), LPC	Greater LPC amplitude to stimulate sexy men in the ovulatory phase
Derntl et al. (2008)^35^	Austria	22 women aged 24.45 ± 3 years (11 in the follicular phase and 11 in the luteal phase)	N (fMRI)	NS	Emotion recognition	3T scanner for amygdala activation (T2 protocol); emotional discrimination task (Vienna Emotion Recognition Tasks (VERT-K)	Greater neural activation for all follicular phase emotions. In particular, negative facial expressions caused greater activation of the amygdala
Zhang, Zhou & Ye (2013)^41^	China	29 women (premenstrual, post-menstrual and ovulation phases); aged 20.6 ± 1.5 years	N (VEP)	NS	Visual electrophysiology	Visual evoked potential; visual latency and amplitude components N1, P2, N2 in Fz, Cz, Pz; P3, LPP in Pz, Oz	Late positive potential (LPP) evokes changes in the premenstrual phase for facial expressions: fear (110-130 ms, 150-190 ms and 300-500 ms), sadness (230-300 ms), and anger (750- 1,000 ms.); lower LPP amplitude in the premenstrual phase
Mareckova et al. (2014)^25^	United Kingdom	20 women (10 in the regular cycle and 10 who used oral contraceptives (menstrual, follicular, mid-cycle and luteal phases); aged 20.44 ± 2.69 years	N (fMRI)	NS	Recognition of emotions	T1-weighted images (T1W), magnetic transfer rate (MTR) and diffusion tensor image (DTI)	Higher level of blood oxygenation (BOLD) in the fusiform area during the mid-cycle phase
Lusk et al. (2015)^37^	Australia	28 women in the follicular phase (23.54 ± 6.6 years); 29 women in the luteal phase (24.41 ± 7.1 years); 27 men	N (VEP)	B	Visual electrophysiology	Visual evoked potential; latency and visual range of P100, P300, N200, N300 and LPP	Early latency in the luteal phase (P1, N1). Early latency for unpleasant images in the menstrual phase in P3 and LPP

N = neuroimaging; NS = not specified; VEP = visual evoked potential; fMRI = functional magnetic resonance imaging; M = monocular; B = neurobehavioral; LPC = late positive complex; LPP = late positive potential.

Table 3 summarizes the articles according to the neurobehavioral methodological criteria (studies with cognitive and behavioral paradigms). A total of 14 articles were found to fit within the criteria that had been established.

**Table 3:** Description of the results according to the neurobehavior method

Authors (year)	Country	Sample (phases of the cycle)	Method	Testing	Visual task	Test / Stimuli	Results
Penton-Voak & Perrett (1999)^30^	United Kingdom	139 women; mean age 20 years (menstrual, follicular and luteal phases)	B	NS	Facial attractiveness	Emotional attractiveness task (female and male face stimuli)	Women showed greater facial attractiveness as facial stimuli in the follicular phase
Penton-Voak et al. (1999)^30^	United Kingdom and Japan	20 women; mean age 21 years (follicular phase and luteal phase)	B	NS	Facial attractiveness	Visual stimuli consisting of male faces	Greater attractiveness to male faces of Caucasians in the follicular phase
Roberts et al. (2004)^47^	United Kingdom and Czech Republic	130 men; 131 women; mean age 25 years	B	NS	Facial attractiveness	Emotional attractiveness task (female face stimuli in the follicular and luteal phases)	Women showed greater facial attractiveness in the menstrual cycle, and higher in the periovulatory than in the luteal phase
Pearson & Lewis (2005)^26^	United Kingdom	50 women of mean age 20 years (12 in the menstrual phase; 13 in the preovulatory phase; 11 in the ovulatory phase; and 14 in the luteal phase)	B	NS	Emotion recognition	Emotion recognition task (Face Bank; Paul Ekman)	Greater accuracy of the fear face in the menstrual phase
Little, Jones, Burt & Perrett (2007)^36^	United Kingdom	31 women aged 20-25 years (in the follicular and luteal phases)	B	NS	Facial attractiveness	Male and female facial stimuli in 2D symmetry	Greater facial attractiveness of symmetrical faces in the follicular phase
Roney & Simmons (2008)^27^	United States	43 women aged 18.36 ± 0.10 years (menstrual phases: follicular, mid-cycle and luteal phases)	B	NS	Facial attractiveness	Visual stimuli consisting of male faces	Greater facial attractiveness of more masculine features in the late follicular phase
Rubinow et al. (2007)^17^	United States	28 women with PMDD (37.9 ± 5.0 years); 27 women without PMDD (33.5 ± 7.0); evaluated in both the follicular and the luteal phases	B	NS	Emotion recognition	Emotional discrimination task	Better discrimination of sad face in the luteal phase for PMDD. Women with PMDD differed significantly from women without PMDD in the luteal phase regarding sad faces
Derntl et al. (2008)^35^	Austria	32 women aged 23.84 ± 3 years (15 in the follicular phase and 17 in the luteal phase)	B	NS	Emotion recognition	Emotional discrimination task (Vienna Emotion Recognition Tasks, VERT-K)	Better performance in the follicular phase; greater confusion in discrimination of negative emotions (anger and disgust) in the luteal phase
Guapo et al. (2009)^32^	Brazil	30 women aged 22.1 ± 2.9 years (11 women in the follicular phase; 9 women in the ovulatory phase; and 10 women in the luteal phase); 10 healthy men	B	NS	Emotion recognition	Prototypes of emotional facial stimuli	Recognition of negative emotions, such as sadness, anger and fear in the follicular phase
Konishi et al. (2009)^29^	Japan	12 women in the menstrual, follicular and luteal phases; aged 22.4 ± 1.1 years	B	NS	Visuospatial memory	Visual memory task using the Baddely model	Higher mental load performance in the luteal phase.
Oberzaucher et al. (2012)^40^	Austria, Czech Republic and Slovakia	10 women and 15 men; aged 23.35 ± 3.1 years)	B	NS	Facial attractiveness	Female facial stimuli in the ovulatory and luteal phases	Greater facial attractiveness of female stimuli in the ovulatory phase. There was no difference regarding the other phases of the menstrual cycle
Puts et al. (2013)^15^	United States	202 women aged 19.6 ± 1.6 years (late follicular and middle luteal phases)	B	NS	Facial attractiveness	Visual stimuli consisting of male faces	Greater facial attractiveness of more masculine features in the late follicular phase (increased estradiol)
Lobmaier, Bobst & Probst (2016)^38^	Switzerland	60 women aged 25.16 ± 5.1 years (2 evaluation phases: ovulatory and luteal)	B	NS	Facial attractiveness	Facial stimuli prototypes	Higher rate of facial recognition during the ovulatory phase
Marcinkowska & Holzleitner (2020)^39^	Poland	102 women aged 28.8 ± 4.6 years	B	NS	Facial attractiveness	Female facial stimuli in the ovulatory and luteal phases	Greater facial attractiveness of female stimuli in the ovulatory phase. There was no difference regarding facial symmetry and sexual dimorphism during the phases of the cycle

B = neurobehavioral; NS = not specified; PMDD = premenstrual dysphoric disorder.

The planning of comparisons, synthesis and interpretation of results were grouped thematically in the discussion, as described below.

## DISCUSSION

Perceptual measurements involve basic and cognitive visual functions that describe how the visual system recognizes and interprets elementary sensory stimuli and/or complex visual scenes.^9^ In this way, visual tests make it possible to investigate how vision detects, processes and recognizes the presence of light and the shape, size and color of visual stimuli, and enable investigation of visual acuity, visual field, evidence of monocular and binocular vision and even image quality.^9^ For visual neuroscience, it is important to describe and understand how these measurements fluctuate with the normal menstrual cycle, i.e. how the peaks and endogenous levels of estradiol and progesterone are related to functional and perceptual changes in vision.^10^ A study of this nature is important even considering that parts of this research are usually conducted with samples of men and women together, which makes it difficult to interpret the results.

### Sample parameters

Most studies have had randomized blinded repeated-measurement research designs with small numbers of samples. However, the research has varied according to the theoretical paradigms and methodological procedure used. For example, studies with psychophysical methods had at least 8 and a maximum of 32 participants, studies with electrophysiology and neuroimaging (for example, visual evoked potential (VEP) and functional magnetic resonance imaging (fMRI)) had samples with a minimum of 11 and a maximum of 30 participants.^11–14^ In studies with neurobehavioral paradigms, the sample size was found to be a minimum of 8 and a maximum number of 202 women.^15^ Even if the sensory threshold is a probabilistic value, its momentary values are normally distributed, except for the sample errors. However, it is important to highlight that an approach based on sampling principles can underestimate hypothesis errors and cross-validation of studies. Therefore, solutions for increasing the sample size should be investigated, so as to address the possibility of increasing the data homogeneity.^16^

### The normal menstrual cycle

The menstrual cycle shows cyclical variations in the levels of various sex hormones. The effects of these hormones are not limited just to the reproductive system but affect other systems too, including the nervous system. All the participants included in the studies had a normal menstrual cycle, but comparative studies between women with a normal menstrual cycle and women who had premenstrual syndrome were also found, along with comparisons with men over periods of time similar to the menstrual cycle.^17–20^

As mentioned earlier, the menstrual cycle can be divided into the follicular, ovulatory and luteal phases.^21^ However, studies have pointed to distinct average phases of the cycle in order to describe behavior during all the phases. For example, the following phases have been described: days 1 to 5 days as the menstrual phase (low levels of progesterone, luteinizing hormone (LH), follicle stimulating hormone (FSH) and estradiol); day 7 as the middle follicular phase (gradual increase in estradiol); days 8 to 11 as the follicular phase (peak estradiol and gradual increases in progesterone, LH and FSH); days 12 to 15 as the ovulatory phase (peak LH and FSH, a gradual increase in progesterone and low levels of estradiol); days 16 to 20 as the luteal phase (increases in progesterone and estradiol and low levels of LH and FSH); day 21 as the middle luteal phase (peak of progesterone and estradiol and low levels of LH and FSH); and days 22 to 28 as the premenstrual phase (decreased levels of progesterone and estradiol and low levels of LH and FSH).^22,23^

These minute divisions suggest that the length of the menstrual cycle may be a relevant indicator for hormonal secretion. Thus, the studies reviewed here were organized as follows: groups of women in the menstrual, follicular, ovular and luteal phases; groups of women in the menstrual, follicular and luteal phases; groups of women in the menstrual, ovulatory and luteal phases; groups of women in the discrete follicular and luteal phases; groups of women in the ovulatory and luteal phases; groups of women in the follicular and middle luteal phases; groups of women in the premenstrual, follicular and ovulatory phases; and a group of women in the follicular phase.^11–15,17–21,23–43^

In addition, hormonal changes that occur during the menstrual cycle influence the body's homeostasis.^31^ Most physiological changes in women occur in the luteal phase: these changes include fluid retention, weight gain, increased energy demand, changes in glucose uptake, lipid profiles, emotional hypersensitivity, generalized pain and changes in dietary habits.^23^

### The psychophysical method

Psychophysical measurements are associated with the sample characteristics and dynamics of the experimental protocols, such as clinical and functional evaluations of the visual system and the methodological characteristics of the research apparatus. In the psychophysical method, the threshold values are usually measured using characteristics of the psychometric function underlying the perceptual performance. In this way, the experimental procedure is adaptable to the physical characteristics of the participants and their responses to sequential attempts to present stimuli.^44^

The results from simulations and experiments involving women were reviewed to assess the usefulness of these adaptive procedures and the special circumstances in which one might be superior to another.^45^ Different articles measured visual tasks that assessed eye movement, visuospatial attention and visual reaction time.^21,23,24,33^ Lastly, there were tests that used the psychophysics toolbox to generate stimuli from circular sinusoidal grids and from Gabor, with the aim of assessing visual sensitivity at different stages of the menstrual cycle.^11,19,20,28,31^ The methodological route that psychophysics offers for carrying out empirical studies, such as noninvasive measurements, has become a perceptual sensory tool of high precision for evaluating thresholds in the phases of the menstrual cycle. Also, from a physiological perspective, this means that these changes have anatomical-physiological connections, with visual pathways integrated with endogenous hormones.

### The neuroimaging method

Functional neuroimaging studies involve experimental baseline conditions that reveal time-space models of visual function. A 3T scanner was used in one study to assess the nature of amygdala activation using a T2 relaxation protocol and the Vienna Emotion Recognition Tasks (VERT-K) was used in another study to verify recognition of facial emotions. In both of these studies, the aim was to understand cognitive functions relating to perception of visual stimuli of emotions, in the follicular and luteal phase.^35^ The recognition of facial emotions was also analyzed by means of T1e-weighted images and diffusion (DTI).^25^ Other models of visual temporal processing were used to measure the latency and amplitude of electroencephalographic components in occipital areas.^14,37,41^ VEP and fMRI are techniques that can be used to understand visual neural events during the normal menstrual cycle.^46^ Together, all these research studies provide temporal and spatial mapping of brain function. On the other hand, all of the studies that evaluated visual functions had the problem of analyzing results that corresponded to measurement of the response to a stimulus after it had been processed by the central nervous system. The response was therefore the sum of the structural visual function and the constructive mental information.

### The neurobehavioral method

This basic and applied research practice uses tests, instruments and behavioral assessment protocols to describe brain functional behavior. Articles in which visual recognition behavior was measured with regard to facial attractiveness of facial stimuli and emotional expressions were found.^15,17,26,27,30,32,35,36,38–40,47^ These studies used a variety of stimuli: for example, prototypes of male and female facial stimuli of different ethnicities at different stages of the menstrual cycle.^39^ In addition to these models, neurocognitive studies were found that evaluated visuospatial memory and visual working memory.^29^

### Visual measurements during the normal menstrual cycle

#### Spatial vision: monocular and binocular measurement

It is important to highlight that some studies used the participants for recording binocular and monocular measurements.^11,18,31,34^ Binocular rivalry occurs when markedly different entries for the two eyes initiate alternations in the perceptual domain between the views of the two eyes.^11^ In psychophysical studies to detect and measure visual thresholds, it has been argued that there are no differences between binocular and monocular measurements.^45^ This hypothesis was confirmed by Giuffrè et al. who found no differences between the left and right eye regarding visual acuity and luminance contrast.^31^ However, Apaydin et al. evaluated 93 eyes of women and highlighted that the lower and upper nasal field of the left eye had greater visual sensitivity in the luteal phase of the menstrual cycle.^34^ These findings were confirmed by Akara et al. in an analysis on lower visual sensitivity of 59 eyes of women also in the luteal phase.^18^ Yılmaz et al. also showed that the mean P100 latency and amplitude were recorded differently binocularly during the menstrual phase.^14^

In another study on binocularity, fluctuations in the gamma-aminobutyric acid (GABA) concentrations during the menstrual cycle of 16 women in the follicular and luteal phase were measured using psychophysical methods, with stimuli of circular sinusoidal grids.^11^ However, no significant changes or trends in menstrual phases were found with regard to the temporal dynamics of ocular dominance. This study pointed to the need for new research perspectives that would investigate GABAergic systems in relation to the dynamics between hormonal steroids and binocular rivalry.

#### Visual acuity

The ability to visually detect something depends on the arrangement and number of photoreceptors per unit area on the retina. In one study, data from the left and right eyes of 15 women in the menstrual, ovulatory, and luteal phases were recorded separately to check their visual acuity performance on the logarithmic minimum angle of resolution (logMAR) scale. No significant differences in acuity values were found in any of the phases.^31^

#### Visual sensitivity

From studies measuring visual sensitivity, it has been suggested that light may be a major factor for the hormonal secretory system. However, these studies have not ascertained any central mechanism for its action. For example, in one study, it was investigated whether hormonal alterations might affect the adaptation to light of four women in the menstrual, follicular and luteal phases.^28^ Variability in visual sensitivity was found at all stages, but there were no specific individual differences. In another study, the effect of light on reproductive hormones was evaluated and the role of photoreception based on melanopsin sensitive to blue (480 nanometers), for mediating the non-visual effects of light, was addressed. That study was carried out among healthy women with measurements in the menstrual, follicular and luteal phases.^43^ The results showed that bright blue light gives rise to secretion of follicle-stimulating hormone in women in the middle follicular phase until the end of the menstrual cycle during the morning, which suggested that a direct functional link between light and the reproductive system existed. The data were seen to be sensitive to the capacity for visual adaptation.

#### Luminance contrast

Luminance contrast sensitivity to stimuli of vertical sinusoidal grids of frequencies 0.5, 1, 2, 4, 8 and 16 cycles per degree of visual angle (cpd) in the menstrual, ovular, and luteal phases was assessed in one study.^31^ No significant differences were found in relation to any of the spatial frequencies tested. Similarly, the luminance contrast of Gabor sinusoidal stimuli was also measured at spatial frequencies of 1, 4 and 16 cpd, in 21 women in the menstrual, ovulatory and luteal phases and in 14 men. It was shown that the sensitivity to visual contrast did not differ according to sex or use of oral contraception. Nor did it vary due to hormonal changes over the course of the menstrual cycle.^20^ It was suggested that changes to the phases of the menstrual cycle did not produce any changes in sensitivity to visual luminance contrast.

#### Visual color

Color discrimination was assessed using the Farnsworth-Munsell 100 test in one study.^31^ The results showed that although all the women in that study could be defined as normal from an ophthalmological point of view, they showed variation in their scores, with better performance in the ovulatory phase and greater numbers of errors of disposition of the records in the menstrual and luteal phases. In addition, there were no significant differences in measurements between the left eye and the right eye. The authors argued that the differences found might be associated with cyclical physiological functions or with the women's psychological state, even if imperfectly expressed. It was concluded that variations in color perception might occur during the menstrual cycle, although the differences were subtle.

#### Visual field

The area of the visual field was measured monocularly in one study, using automated perimetry with a short wavelength, among 48 eyes of healthy women and 45 eyes of women with diabetes mellitus, who were in the follicular and luteal phases of the menstrual cycle.^34^ To highlight the results, the authors pointed out that the achromatic visual field tests were not statistically different between diabetic women and the control group. However, the mean values of the temporal retinal region were significantly lower in the luteal phase than in the follicular phase in the diabetic women. In another study, standard achromatic perimetric analysis was performed using automated short-wavelength perimeters of monocular shape, among 59 healthy women in the follicular and luteal phases, and the results were compared with those from 54 men. Unlike in the previous study, a reduction in the average value in the automated perimeter field was found for short wavelength in the luteal phase.^18^

In the above two studies,^18,34^ the authors were of the opinion that the hormonal behavior that occurs in the luteal phase correlates with a reduction of loss of sensation in the visual field. However, further clinical trials relating to synchronization and visual campimetry should be conducted in order to provide more accurate information.

#### Spatial orientation

The perception of verticality was evaluated in one study through the binocular visual acuity of 30 women in the follicular phase and 24 women in the luteal phase, and their results were compared with those of 30 men over the same time periods. The subjects had been matched previously with regard to the menstrual cycle using a mechanical rod and frame test system.^19^ A higher proportion of vertical positioning errors was found among the women in the middle luteal phase than among the men or among the women in the follicular phase, which suggested that the phase was associated with the perception of verticality. Similarly, the spatial orientation of 24 women in the menstrual and luteal phases and 14 men was assessed in another study using a visual line bisection task to investigate functional brain asymmetry.^23^ It was shown that high levels of estradiol during the luteal phase were related to decreased hemispheric asymmetry of spatial attention, with greater bias towards the left hemisphere.^19^ These findings contribute to the idea that the luteal phase can present strong evidence of fluctuations in orientation and spatial attention.

#### Eye movement

The optokinetic function of 12 women in the menstrual, follicular, half-cycle and luteal phases was measured in one study. The authors suggested that there was no difference in the pattern of eye movement. The results from that study demonstrated that hormonal changes that occur throughout the menstrual cycle had no significant effect on the speed of fixation movements.^24^

#### Visual electrophysiology

Several studies have investigated the electrophysiological changes in visual functions that occur during the menstrual cycle of women. For example, pattern-reversal visual evoked potentials (PRVEPs) were investigated among 30 healthy women in the menstrual, follicular, ovulatory and luteal phases.^14^ Although the results were not statistically significant, there was greater mean amplitude of the P100 during the ovulatory phase in relation to all other phases. Thus, the P180 latency was statistically lower in the follicular phase than in the luteal phase. In another similar study, changes to the potential relating to social stimulus events were investigated among women in the menstrual, ovulatory and luteal phases.^13^ Those results also showed an increase in the ovulatory phase in comparison with the luteal phase, for late positive complex (LPC) stimulus processing indicators (500-700 millisecond). According to those authors, the reason for those variations in amplitude and latency was the increase in estrogen in the follicular phase. They reported that estrogen caused a decrease in the time taken for visual transmission, thereby increasing the sensitivity of receptors in the dopamine optical pathways.

Visual amplitude and latency in the processing of recognition of modulated facial expression during the phases of the menstrual cycle was measured in one study.^41^ Visual evoked potentials (VEPs) were evaluated among 29 healthy women in the premenstrual, post-menstrual and ovulation phases, in relation to recognition of different emotional expressions. The authors of that study pointed out that the late positive potential (LPP) (750 to 1,000 milliseconds) was affected in the central parietal area (Pz) and central occipital area (Oz) by the menstrual cycle, for all facial expressions. Thus, the amplitude in the ovulation phase was greater than in the premenstrual phase, which meant that there was a positive correlation between the amplitude of the LPP and performance in facial expression recognition during the ovulation phase. In addition, the amplitude of P3 (300-500 milliseconds) in Oz in the ovulation phase was marginally significant, in comparison with the premenstrual phase and the post-menstrual phase. These greater amplitudes occurred for fear-based stimuli, in comparison with stimuli consisting of neutral and happy faces.

Similarly, in another study, VEPs were investigated by means of visual stimuli among 28 women in the follicular phase and 29 women in the luteal phase. The authors used pleasant and unpleasant image stimuli with low and high intensities and found greater amplitude of P3 and LPP in the luteal phase for unpleasant images, in relation to all other stimulus conditions.^37^

These studies pointed towards cortical processing of visual stimuli and found significant evidence showing that VEPs have a role in improvement of the automatic visual process of emotional stimuli associated with the luteal menstrual phase, especially in relation to unpleasant stimuli and those with a negative emotional charge.

#### Emotion recognition

Recently, some studies have evaluated neural modulation in relation to recognition of facial emotion according to the phase of the menstrual cycle and sex hormones. For example, the mediating role of the menstrual cycle stage was evaluated with regard to recognition of emotional expressions among 50 women in the menstrual, pre-ovulatory, ovulatory and luteal phases.^26^ The authors of that study found that there was greater accuracy regarding fearful faces during the menstrual phase and suggested a hypothesis regarding estrogen levels in the coding of facial stimuli. The fact that fear recognition is affected differently according to the phase of the cycle suggested that there was neural processing distinct from fear.

A comparative study on healthy women and women with premenstrual dysphoric disorder (PMDD) also investigated emotional recognition in order to determine emotional processing errors in the follicular and luteal phases of the menstrual cycle.^17^ In that study, there were no differences in emotional recognition among healthy women but it was noted that women with PMDD showed better recognition of negative faces during the luteal phase. According to the authors of that study, this negative bias might contribute to generation of negative mood states during the luteal phase and might suggest the presence of dysfunction in brain regions whose activity mediates recognition of emotion in facial expression. Those findings corroborated the results from electrophysiological investigations.^13,41^

The accuracy of facial emotion recognition was evaluated among 40 healthy volunteers.^32^ The results showed that women in the follicular phase had greater accuracy in recognizing emotions of sadness and fear than did women in the luteal phase. Similar results were found using fMRI techniques. fMRI measurements with T1 relaxation were made among 20 women at different stages of the cycle and higher levels of blood oxygenation (BOLD) were found in the right spindle area among women taking oral contraceptives than among women who did not use contraceptives during the follicular phase.^25^ In addition, the accuracy of emotional recognition was investigated among 22 women (11 in the follicular phase and 11 in the luteal phase) using fMRI T2 relaxation measurements in relation to amygdala activation, and greater neural activation was found for negative emotions such as fear and disgust in the follicular phase.^35^ Similar results were found through a neurobehavioral analysis among 35 women during the follicular and luteal phases.^35^ Thus, these authors suggested that there was a significant negative correlation between progesterone levels and performance in emotion recognition, such that there was greater accuracy of socioemotional influence with lower levels of progesterone.

#### Visual attractiveness

Facial features can serve as a clue to social judgments of multiple human features. In one study, female facial stimuli were used in the ovulatory and luteal phases to ascertain the symmetry of facial attractiveness. It was suggested that the greatest facial attractiveness of women was found in the most fertile phase of the menstrual cycle.^39^ In another study, an emotional attractiveness task regarding stimuli consisting of female faces, conducted during the follicular and luteal phases, showed that the attractiveness perceived in the women's faces varied over the course of the menstrual cycle and was greater in the ovulatory phase than in the luteal phase.^47^

Facial attractiveness was evaluated among 60 women who were presented with stimuli consisting of female faces.^38^ They did not find particular attractiveness in faces in the late follicular or luteal phase, but found higher attractiveness during the ovulatory phase. It is important to note that women with high levels of estradiol are more likely to choose the faces of women who are also in the ovulatory phase. In another study, the preferences of men and women for female faces during the ovulatory and luteal periods were investigated and it was shown that there was greater preference for faces in the ovulatory phase. According to the authors of that study, the preference for facial attractiveness in the ovulatory phase correlated with the hormonal fertility state.^40^

Stimuli of male and female faces were used in a study aimed at measuring the facial attractiveness of women in the follicular and luteal phases. It was found that there was a higher attractiveness rate in the follicular phase of the menstrual cycle than in the menstrual and luteal phases.^30^ Twenty women in the follicular phase and luteal phase were asked to rate the attractiveness of male faces of Caucasian and Japanese ethnicity.^30^ There was greater preference for the faces of Caucasian men during the follicular phase than during the luteal phase. Likewise, the facial attractiveness of 31 women in the follicular and luteal phases was measured through the symmetry of male faces. It was shown that women preferred more symmetrical faces during their peak fertility.^36^ In line with previous studies, 43 women in the menstrual, follicular, mid-cycle and luteal phases were evaluated and greater attractiveness towards male faces was found during the late follicular phase.^27^ In another study, women were more attracted to faces with a greater trait of masculinity when they were in the late follicular phase, due to the gradual increase in estradiol.^15^

The converging evidence suggests that the menstrual cycle, and, therefore, hormone levels, can affect emotional behavior, in particular recognition of facial emotions. While universal preferences may exist for particular characteristics in populations, it appears that there are several individual differences in facial preferences that are dependent on the individual's situation, individual characteristics and hormone levels.^27,38,39^

#### Visual reaction time

Reaction time is defined as the time interval between application of a stimulus and an appropriate voluntary response from the individual. The influence of the menstrual cycle on the visual (TRV) and auditory (TRA) reaction times of 30 women was investigated and it was shown that TRV and TRA were more prolonged in the luteal phase than in the follicular phase.^21^ These results could be attributed to the fluctuating levels of female sex hormones. In another study, the TRV and TRA of 50 women in the menstrual, ovulatory and luteal phases were measured using presentation of stimuli such as flashes of light and sound.^33^ It was concluded that TRV and TRA were longer during the menstrual phase and shorter during the luteal phase of the menstrual cycle. The data were contradictory, but the authors of that study pointed out that the prolonged reaction times during the menstrual phase were attributable to the decrease in driven reaction times, due to increases in the levels of fluids and electrolytes, and that faster reaction times were due to progesterone, which negates the ability of estrogen to cause a delay in driven reaction time.

#### Visuospatial cognitive function

Working memory is a faculty of the mind that enables multiple tasks to be performed simultaneously, in parallel. It involves controlling the distribution of concentration, with individual differences in attention span. A study examined visuospatial cognitive functions in the follicular, luteal and menstrual phases of 12 healthy women, with regard to visual working memory.^29^ The authors showed that the greatest mental workload and stress was perceived in the luteal phase.

## CONCLUSIONS

The results from the studies presented were inconclusive, but these inconsistencies may have been related to lack of experimental control and failure to assess the perimetry of the phases of the menstrual cycle. These findings continue those of Guttridge (1994), although it is difficult to draw specific conclusions about the performance of visual functions during the phases of the normal menstrual cycle. Therefore, further research is needed in order to clarify the sensory and perceptual mechanisms involved in the menstrual cycle, especially with regard to facial recognition and attractiveness processes. It is noteworthy that while the study by Guttridge (1994) was aimed more towards optical measurements, our study sought to understand neurological and behavioral perceptual processes. However, the role of hormones and the phases of the menstrual cycle in the psychophysical, neurobiological and behavioral traits of visual functions could be observed, and specifically in relation to the follicular and luteal phases.

Visual detection fluctuates significantly during the menstrual cycle. Observations relating to visual processing during the menstrual cycle may explain the physiological, cognitive, behavioral and social modulations of healthy women. In addition, a good proportion of visual measurements (with some exceptions, such as visual acuity) are dynamic and can naturally fluctuate between one measurement and another. Perhaps for this reason, it is common to use repeated measurements. In addition, yet other variables (for example, circadian rhythms) that can affect visual measurements generally do not appear in studies.

However, correlation of physiological and clinical psychological data with visual data from the phases of the menstrual cycle can provide a contribution, as important biological and behavioral markers of human vision. In addition, this can facilitate knowledge of the sensory and perceptual biological rhythms of vision during the physiological changes of the menstrual cycle.
